# Galectin-3 Plays an Important Role in Preterm Birth Caused by Dental Infection of *Porphyromonas gingivalis*

**DOI:** 10.1038/s41598-018-21072-y

**Published:** 2018-02-12

**Authors:** Mutsumi Miyauchi, Min Ao, Hisako Furusho, Chanbora Chea, Atsuhiro Nagasaki, Shinnichi Sakamoto, Toshinori Ando, Toshihiro Inubushi, Katsuyuki Kozai, Takashi Takata

**Affiliations:** 1 Department of Oral and Maxillofacial Pathobiology, School of Biomedical and Health Sciences, Hiroshima University, Hiroshima, 734-8553 Japan; 2 Department of Pediatric Dentistry, Institute of Biomedical and Health Sciences, Hiroshima University, Hiroshima, 734-8553 Japan; 3 Present Address: Laboratory of Oral Connective Tissue Biology, NIAMS/NIH, USA; 4 Human Genetics Program, Institute of Sanford Burnham Prebys Medical Discovery, 10901 North Torrey Pines Road, La Jolla, CA 92037 USA; 5 Present Address: Laboratory of Oral Connective Tissue Biology, National Institute of Arthritis and Musculoskeletal and Skin Diseases, National Institutes of Health, 9000 Rockville Pike, Building 50, Room 4120, Bethesda, MD 20892 USA; 6 Present Address: University of California, San Diego, Moores Cancer Center, 3855 Health Sciences Dr., La Jolla, CA 92093-1503 USA; 7 Department of Orthodontics and Dentofacial Orthopedics, Graduate School of Dentistry, Osaka University, 1-8 Yamada-Oka, Suita, OSAKA 565-0871 Japan

## Abstract

Dental infection is risk for preterm birth (PTB) through unclear mechanisms. We established a dental infection-induced PTB mouse model, in which *Porphyromonas gingivalis* (*P.g*.) induced PTB by 2 days. We analysed pathogenic factors contributing to PTB and their effects on trophoblasts *in vitro*. TNF-α, IL-8, and COX-2 were upregulated in *P.g*.-infected placenta. Galectin-3 (Gal-3), an immune regulator, was significantly upregulated in placenta, amniotic fluid, and serum. *In vitro*, *P.g.-*lipopolysaccharide (*P.g.-*LPS) increased TNF-α and Gal-3 in trophoblasts via NF-κB/MAPK signalling. Gal-3 inhibition significantly downregulated *P.g.-*LPS-induced TNF-α production. TNF-α upregulated Gal-3. Gal-3 also increased cytokines and Gal-3 through NF-κB/MAPK signalling. Moreover, Gal-3 suppressed CD-66a expression at the maternal-foetal interface. Co-stimulation with Gal-3 and *P.g.-*LPS upregulated cytokine levels, while Gal-3 plus *Aggregatibacter actinomycetemcomitans (A.a.)-* or *Escherichia coli (E. coli)-*LPS treatment downregulated them, indicating the critical role of Gal-3 especially in *P.g*. dental infection-induced PTB. *P.g.-*dental infection induced PTB, which was associated with Gal-3-dependent cytokine production. New therapies and/or diagnostic systems targeting Gal-3 may reduce PTB.

## Introduction

Preterm birth (PTB) is a leading cause of infant morbidity and mortality in new-borns. PTB is characterized as delivery at <37 weeks of gestation^1^. The frequency of PTB is approximately 5% in Japan and several European countries, 12–13% in the United States, and 18% in some African countries^2^. PTB is associated with 75% of perinatal mortality and >50% of the long-term morbidity, such as cerebral palsy, bronchopulmonary dysplasia, intraventricular haemorrhage, and necrotizing enterocolitis^3^. Maternal or fetal indications account for 30% of PTB cases (so-called indicated PTB), while spontaneous PTB with or without premature preterm rupture of the membranes accounts for 25% or 45% of PTB cases, respectively^1^. Causes of spontaneous PTB include infection, inflammation, vascular disease, and uterine over-distention. Infections and infection-driven inflammatory responses are considered to be the leading causes of spontaneous PTB^4^. It is well accepted that various stimulatory factors such as proinflammatory cytokines, chemokines, and prostaglandins trigger the onset of labour near the end of pregnancy by inducing cervical ripening and myometrium activation. These stimulatory factors can be induced by intrauterine infection and then lead to PTB^5^. It is reported that intrauterine infection increases the production of prostaglandins, TNF-α, IL-6, and IL-8, which leads to more frequent and intense uterine contractions with consequential PTB^6,7^.

Periodontal disease is a chronic infectious disease, mainly caused by gram-negative periodontal pathogens, which can enter the blood circulation and disseminate throughout the entire body. Periodontitis is considered a risk factor of systemic diseases such as cardiovascular disease and diabetes mellitus^8,9^. In 1996, the results from a case-control study performed by Offenbacher *et al*. suggested that maternal periodontal disease was associated with a 7.9-fold increased risk for PTB/low birth weight^10^. In pregnant women with a diagnosis of threatened premature labour, periodontal pathogens were detected both in the periodontal pocket and in the amniotic fluid^11^. In addition, the presence of periodontal pathogens in the placenta was confirmed in cases of chorioamnionitis where PTB was also indicated^12^. However, the underlining mechanisms of periodontitis-induced PTB remain unclear.

Several experimental studies have revealed that *Porphyromonas gingivalis* (*P.g*.), a main periodontal pathogen, can invade the placental-fetal barrier to induce pregnancy complications in rodents and rabbit animal models^13–15^. However, the bacteria-delivery systems used, such as intravenous or peritoneal injection, the subcutaneous chamber model, and oral-gavage are quite different from the natural infection route used by *P.g*. to infect patients. Recently, we established a dental infection-induced PTB mouse model that showed long-term low-grade inflammation, which ideally imitates the clinical condition of pregnant women with periodontitis. In the model, *P.g*. dental infection induced PTB (by an average of 2 days). *P.g*. originating from a dental infection was detected in placental tissue by immunohistochemistry and PCR. Moreover, in *P.g.-*infected placenta, upregulation of inflammatory mediators such as TNF-α and COX-2 (inducible synthetase of prostaglandins) was observed, which can promote the initiation of labour^16^.

In the present study, our aim was to clarify PTB-related molecules that can differentiate PTB from term birth (TB), using our established animal model. We identified an interesting immune-regulatory molecule, Galectin-3 (Gal-3), which was upregulated in *P.g*.-infected placental tissue compared control placentas. Moreover, the underlining mechanisms, in which Gal-3 induces PTB, were studied a using human trophoblast cell line.

## Results

### Dentally Applied *P.g*. Induced Defects in Mouse Placental Tissue through Inflammatory Mediators

**Figure 1:**
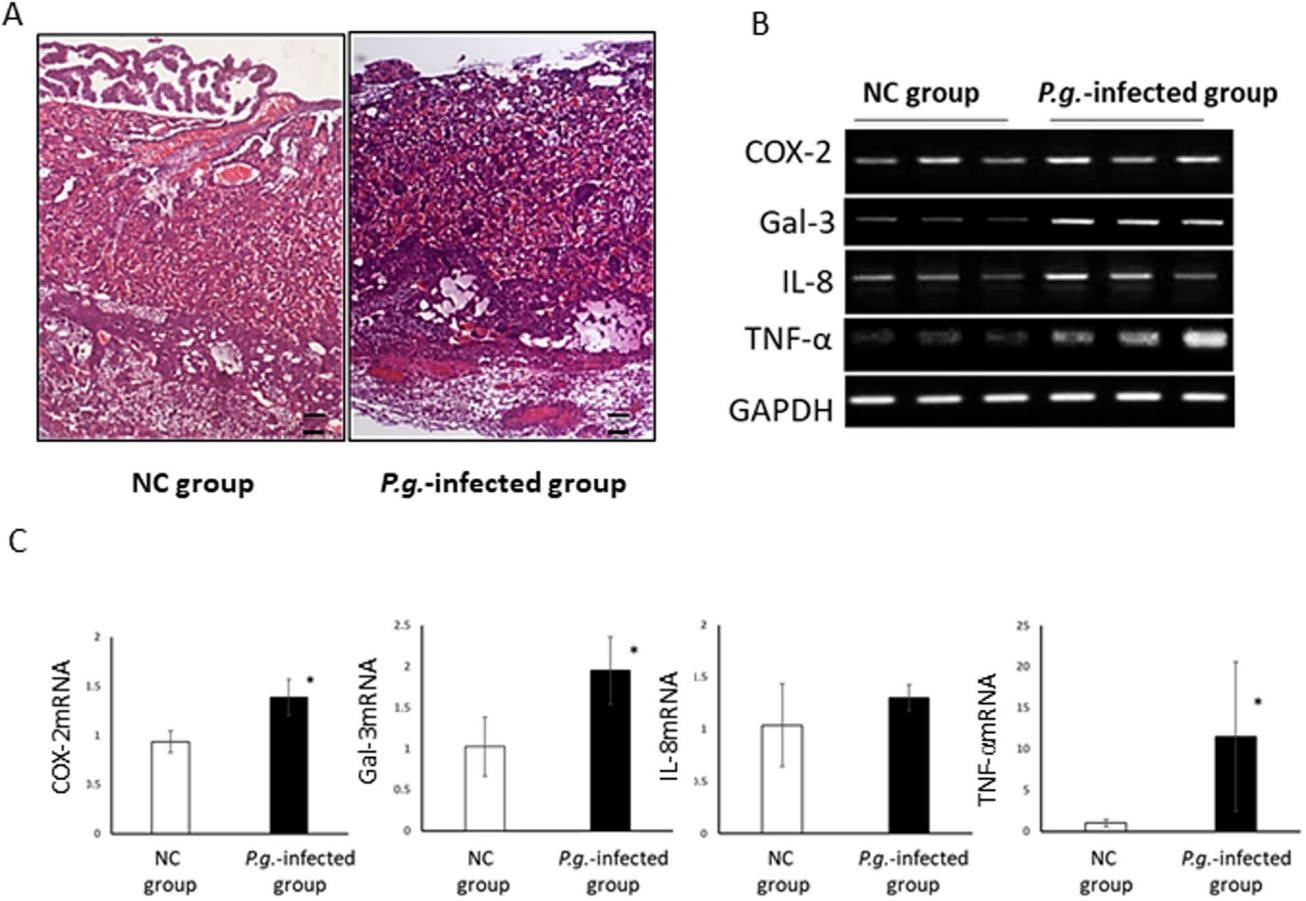
Dentally applied *P.g*. induced tissue damages and upregulation of PTB related cytokines in mouse placental tissues. (**A**) Representative histological findings of gd15-placental tissue of the NC group and the *P.g*.-infected group by H&E staining (n = 6, each group). The amniotic membrane degenerated, and trophoblasts and endothelial cells were necrotic in the *P.g*.-infected group. Scale bars; 100 μm. (**B**) Representative COX-2, Gal-3, IL-8, and TNF-α mRNA expression data in placental tissues in the NC and *P.g*.-infected groups. GAPDH expression was detected as an internal control. (**C**) Quantitative real time PCR analysis of COX-2, Gal-3, IL-8, and TNF-α. *p < 0.05 significant difference between NC and *P.g.-*infected group.

We confirmed that histological damage occurred in *P.g*.-infected placental tissue, as we reported previously^16^. In normal placentas of gd15, no regressive changes were observed, and the decidua was firmly attached to the uterus. However, in *P.g.-*infected placental tissue, the amniotic membrane was degenerated, and necrosis was seen in the labyrinth and decidua (Fig. 1A). COX-2, Gal-3, and TNF-α mRNA expressions were significantly upregulated in *P.g*.-infected placental tissue (*p < 0.05) (Fig. 1B and C).

### Dentally Applied *P.g*. Increased Gal-3 Expression in the Placenta, Amniotic Fluid, and Maternal Serum

**Figure 2:**
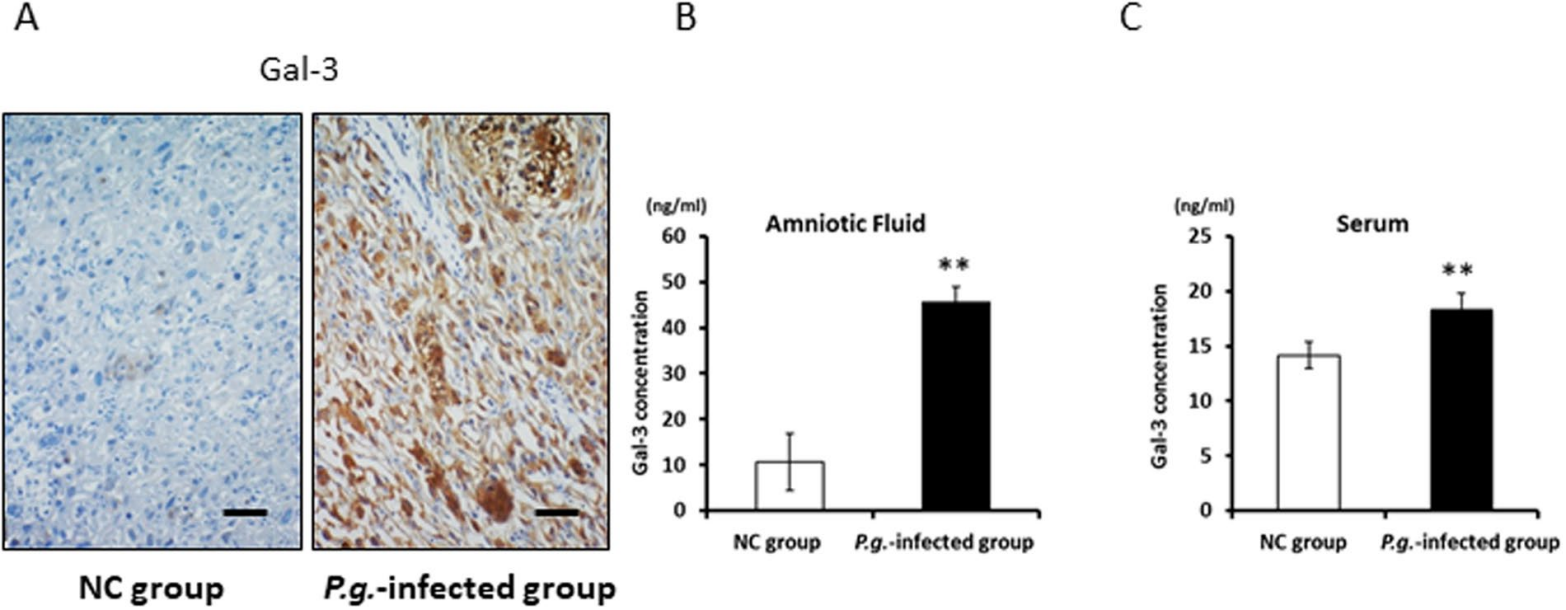
Gal-3 expression in the *P.g*.-infected group was upregulated. (**A**) Gal-3 immunohistochemistry in gd15-placental tissue of the NC group and *P.g*.-infected group. In addition to macrophages, trophoblasts strongly expressed Gal-3. Scale bars, 100 μm. ELISA analysis of the Gal-3 concentration in amniotic fluid (**B**) and maternal serum (**C**) from the NC group and *P.g.-*infected group (n = 6, each). Data are presented as the mean ± SD. **P < 0.01; Student’s t-test.

Because the expression of Gal-3 mRNA, an important immune regulatory molecule, was prominently upregulated in *P.g*.-infected placental tissue (Fig. 1B,C), Gal-3 immunoexpression was examined to confirm its production in the placenta. Interestingly, ubiquitous Gal-3 staining was detected in only *P.g*.-infected placenta of 15gd mice. Gal-3 was expressed not only in inflammatory macrophages, but also in trophoblasts (Fig. 2A).

Because intensive Gal-3 production in *P.g*.-infected placenta may induce elevated Gal-3 levels in the amniotic fluid and maternal serum, we examined Gal-3 levels in both locations. As expected, Gal-3 concentrations in the amniotic fluid (Fig. 2B) and maternal serum (Fig. 2C) were significantly increased in the *P.g.-*infected group, compared to those in the NC group (**P < 0.01).

### Gal-3 Plays an Important Role in the Inflammatory Activity of *P.g*.-LPS

It is well known that Gal-3 is one of important products from macrophages with LPS stimulation and is a key immunoregulator to produce inflammatory cytokines^17^.

**Figure 3:**
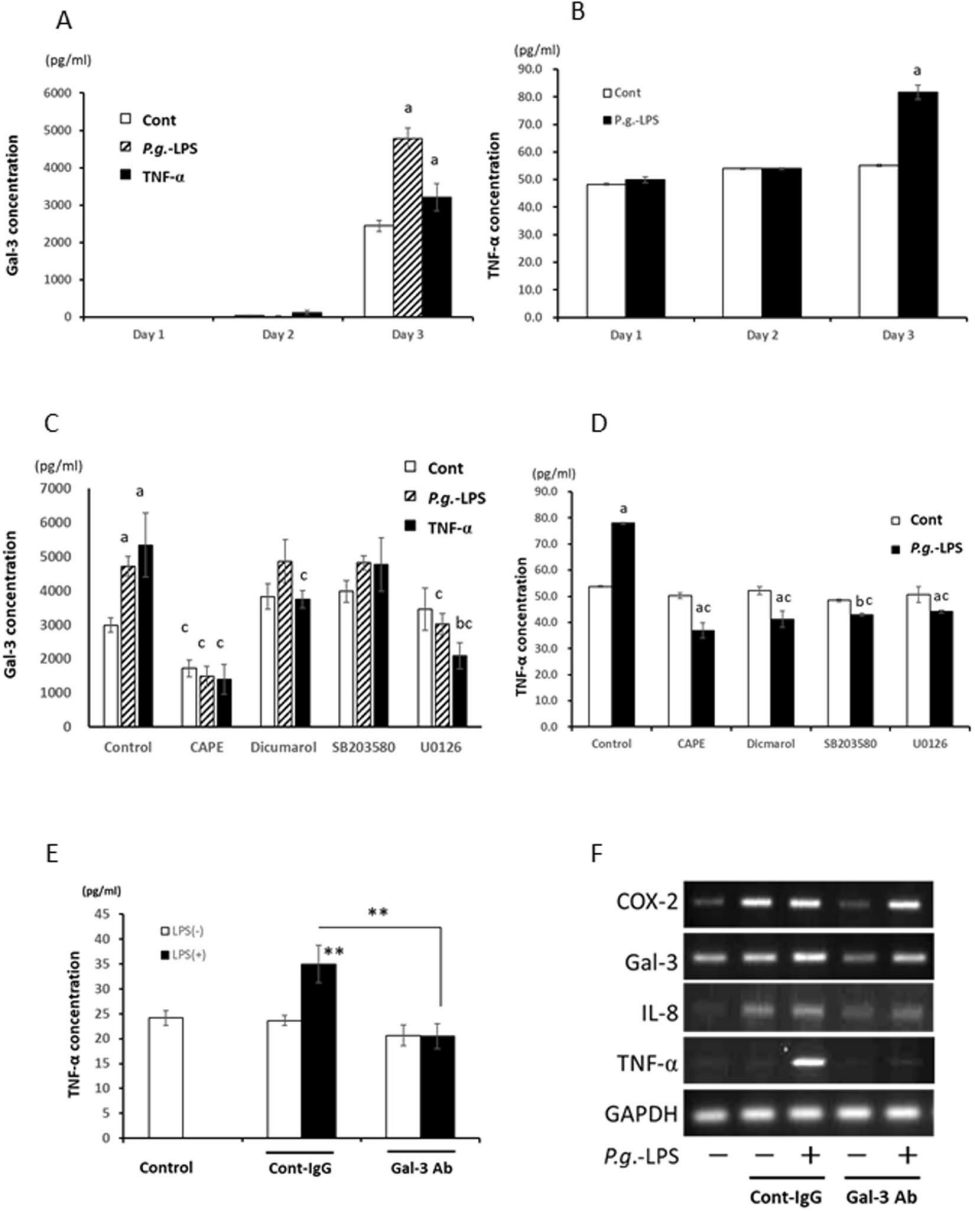
Gal-3 played an important role in the Inflammatory activity of *P.g*.-LPS. (**A**) Gal-3 levels in culture media were analysed by ELISA. *P.g.-*LPS (1 µg/ml) significantly induced Gal-3 in trophoblasts (HTR-8 cells) at 3 days. HTR-8 cells incubated with rhTNF-α (10ng/ml) also significantly produced Gal-3 at 3 days after incubation. (**B**) TNF-α levels produced from HTR-8 cells with *P.g*.-LPS (1 μg/ml) were measured. After 3 days, TNF-α production was significantly upregulated. (**C**,**D**) HTR-8 cells were pre-treated with or without CAPE (1 μg/ml) for 4 h, pre-exposed to dicumarol/SB203580/U0126 (10 μM) for 30 min, and then grown with or without *P.g.-*LPS (1 µg/ml) or rhTNF-α (10ng/ml) for 3 days. The Gal-3 or TNF-α concentration in the culture media was analysed by ELISA. CAPE, and U0126 inhibited Gal-3 production induced both by *P.g.-*LPS and rhTNF-α (**C**). All inhibitors used were significantly reduced TNF-α secretion from HTR-8 cells at 3 days after LPS-stimulation. Data are presented as the mean ± SD. a and b: significant difference (P < 0.01, p < 0.05) between untreated control, c: significant difference (P < 0.01) between *P.g*.-LPS/rhTNF-α stimulated and control group; 1-way ANOVA. (**E**,**F**) HTR-8 cells were treated with *P.g*.-LPS (1 μg/ml) with or without a Gal-3 blocking antibody (clone M3/38) or rat control IgG for 3 Days. Gal-3 antibody significantly reduced *P.g.-*LPS induced TNF-α production (**E**). COX-2, IL-8, and TNF-α mRNA-expression levels were examined. GAPDH expression was detected as an internal control (**F**). Data are presented as the mean ± SD. **P < 0.01; 1-way ANOVA. The experiments were performed at least 3 times with similar results.

*P.g*.-LPS upregulated Gal-3 secretion from trophoblasts (HTR-8 cells: a; p < 0.01) at day 3 (Fig. 3A) as well as macrophages (THP-1 cells: p < 0.01) at day 1, 2 (Supplement Fig. 1A) and endothelial cells (Huh-1 cells: p < 0.01) at day 3 (Supplement Fig. 1B). At 3 days after *P.g*.-LPS stimulation, TNF-α significantly secreted from HTR-8 cells (a; P < 0.01; Fig. 3B). To examine the effect of TNF-α produced by *P.g*.-LPS stimulation on Gal-3 production, HTR-8 cells were incubated with rhTNF-α and Gal-3 secretion was examined in the culture medium. Significantly increased Gal-3 production was detected after 3 days rhTNF-α-stimulation (a; p < 0.01, Fig. 3A).

To clarify the signaling pathways relating to Gal-3 production, inhibitors were used. CAPE (an NF-κB inhibitor) and U0126 (an ERK inhibitor) suppressed *P.g.-*LPS and rhTNF-α induced upregulation of Gal-3 secretion (c; p < 0.01, Fig. 3C). Inhibition of NF-κB signalling significantly reduced constitutive secretion of Gal-3 in HTR-8 cells (c; p < 0.01, Fig. 3C).

All examined NF-κB and MAPK signalling inhibitors completely downregulated *P.g*.-LPS induced TNF-α production (c; p < 0.01, Fig. 3D). Moreover, the inflammatory activity of *P.g*.-LPS was restricted by treatment with a Gal-3 blocking antibody. *P.g*.-LPS-induced TNF-α secretion by HTR-8 cells was significantly reduced to control level after Gal-3 antibody treatment (**P < 0.01, Fig. 3E). Gal-3, IL-8, and TNF-α gene-expression levels were down-regulated by Gal-3 blocking antibody but not COX-2 (Fig. 3F).

### Gal-3 Activated the JNK and NF-κB Pathways, Playing an Important Role in the Inflammatory Activity of *P.g*.-LPS

**Figure 4:**
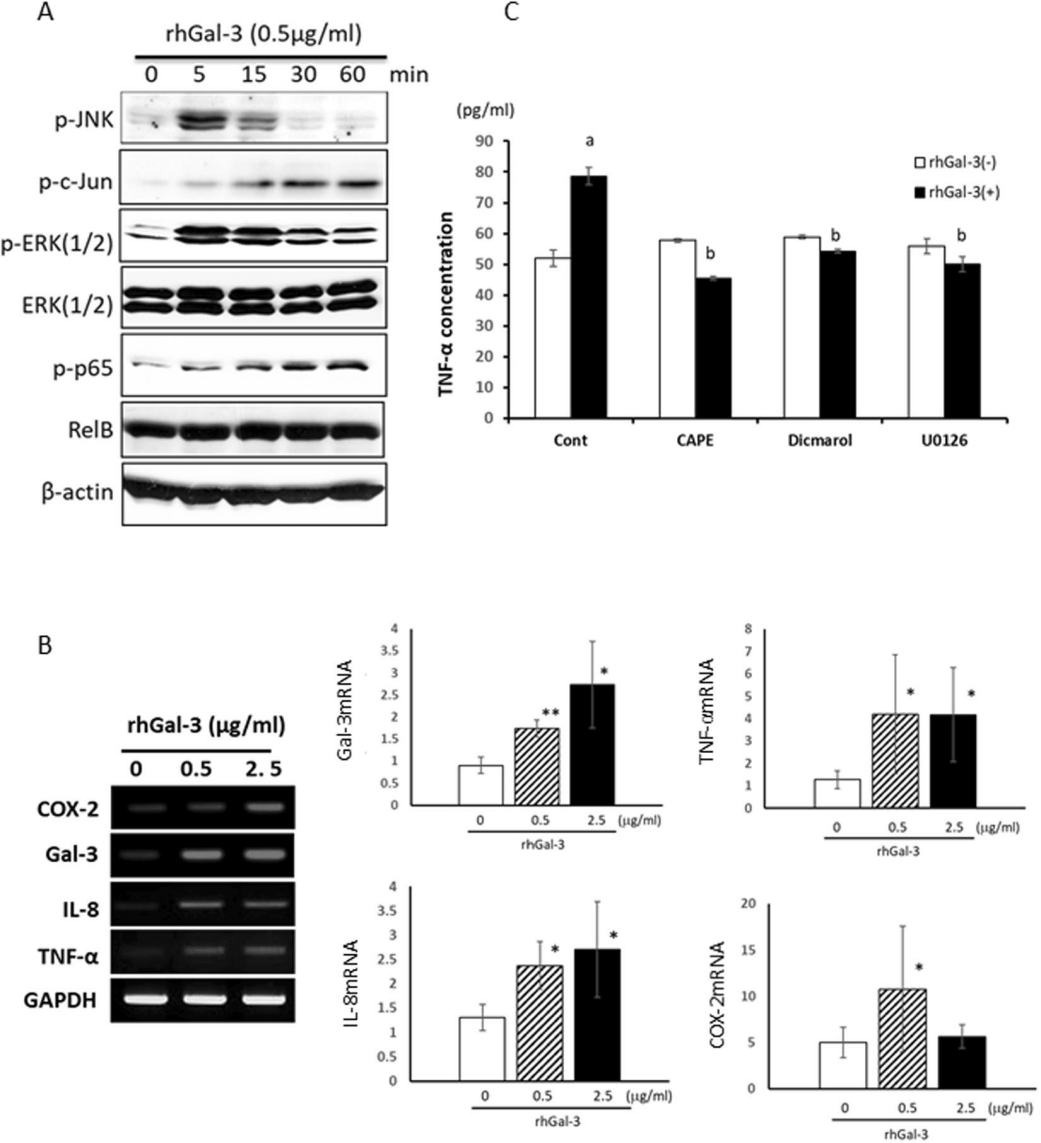
Gal-3 activated the JNK and NF-κB pathways and played an important role in the inflammatory activity of *P.g*.-LPS. (**A**) Trophoblasts (HTR-8 cells) were stimulated with rhGal-3 (0.5 μg/ml), and cell lysates were examined by immunoblot analysis. Phosphorylation of JNK, c-Jun, ERK and p65 was evident. (**B**) HTR-8 cells were stimulated by different concentrations of rhGal-3 (0, 0.5, 2.5 μg/ml) for 48 h. The mRNA-expression levels of COX-2, Gal-3, IL-8, and TNF-α were analysed by PCR and real time PCR. The expression of cytokines were markedly upregulated. (**C**) TNF-α production from rhGal-3 (1 μg/ml)-stimulated HTR-8 with or without CAPE (1 μg/ml) for 4 h, pre-exposed to dicumarol/U0126 (10 μM) for 30 min were measured by ELISA after 2 days. Data are presented as the mean ± SD. *P < 0.05; **P < 0.01; 1-way ANOVA. The experiments were performed at least 3 times with similar results.

rhGal-3 treatment activated the phosphorylation of JNK, c-Jun, ERK and p65 within 5 min. p-JNK and pERK peaked at 5 min and lasted till 15 min. On the other hand, p-c-JUN and p-p65 gradually upregulated in 60 min (Fig. 4A). Similarly, rhGal-3 induced Gal-3 mRNA expression in HTR-8 cells (Fig. 4B). rhGal-3 also significantly upregulated the mRNA expression of inflammatory mediators such as COX-2, IL-8, and TNF-α (Fig. 4B).

To clarify the signaling pathways relating Gal-3-induced TNF-α production, inhibitors were used. Inhibition of NF-κB, ERK and JNK signaling pathway significantly reduced Gal-3-induced TNF-α secretion (Fig. 4C).

### Gal-3 Inhibition Using a Blocking Antibody or Gene Knockdown Increased CD66a Expression

**Figure 5:**
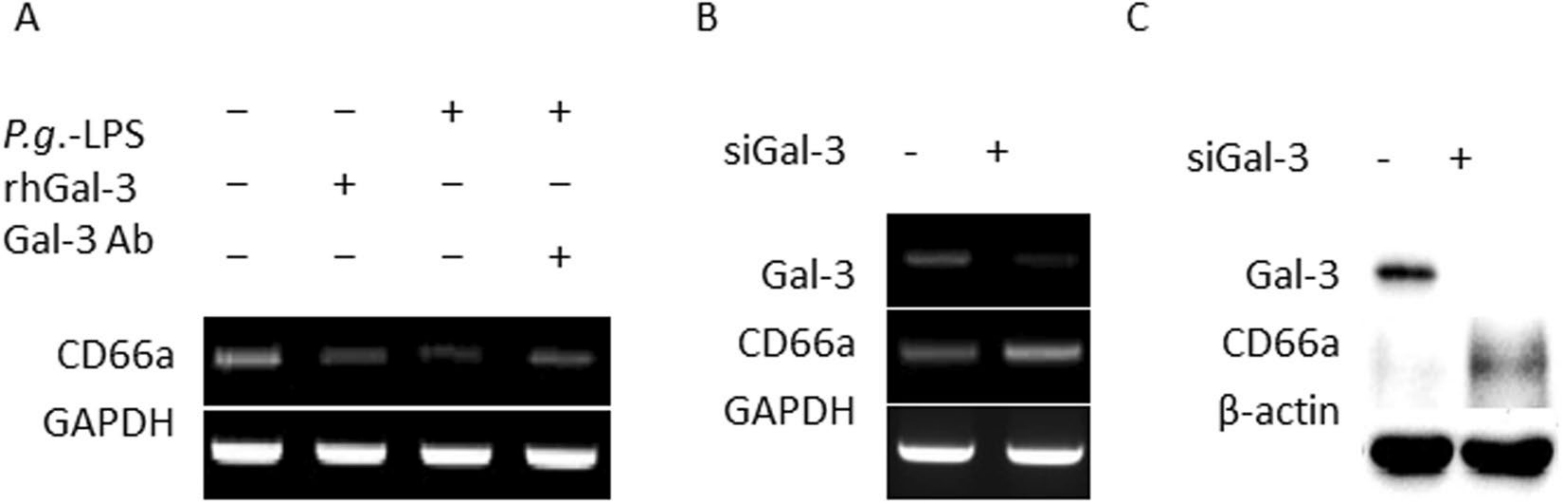
Gal-3 inhibition via a blocking antibody or gene knockdown increased CD66a expression in trophoblasts. (**A**) Trophoblasts (HTR-8 cells) were stimulated for 48 h with rhGal-3, *P.g.-*LPS, or a Gal-3 blocking antibody together with *P.g.-*LPS. CD66a mRNA expression was examined after exposure to *P.g*.-LPS and rhGal-3 with or without the Gal-3 antibody. (**B**,**C**) siRNA-mediated Gal-3 knockdown was performed for 48 h. CD66a expression at both the mRNA and protein levels. GAPDH or β-actin expression was detected as an internal control. The experiments were performed at least 3 times with similar results.

CD66a is an adhesion molecule that is normally expressed in trophoblasts, functioning as an important adhesion mediator at the maternal-foetal interface^18^ and a Gal-3 receptor on neutrophils^19^. Both rhGal-3 and *P.g*.-LPS treatment decreased CD66a mRNA expression, and a Gal-3 blocking antibody rescued CD66a downregulation induced by *P.g.-*LPS (Fig. 5A). Moreover, siRNA-mediated Gal-3 knockdown caused CD66a upregulation at both the mRNA (Fig. 5B) and protein levels (Fig. 5C).

### Gal-3 could not Bind to *P.g*.-LPS and Both Molecules Synergistically Upregulated the Expression of Inflammatory Mediators

**Figure 6:**
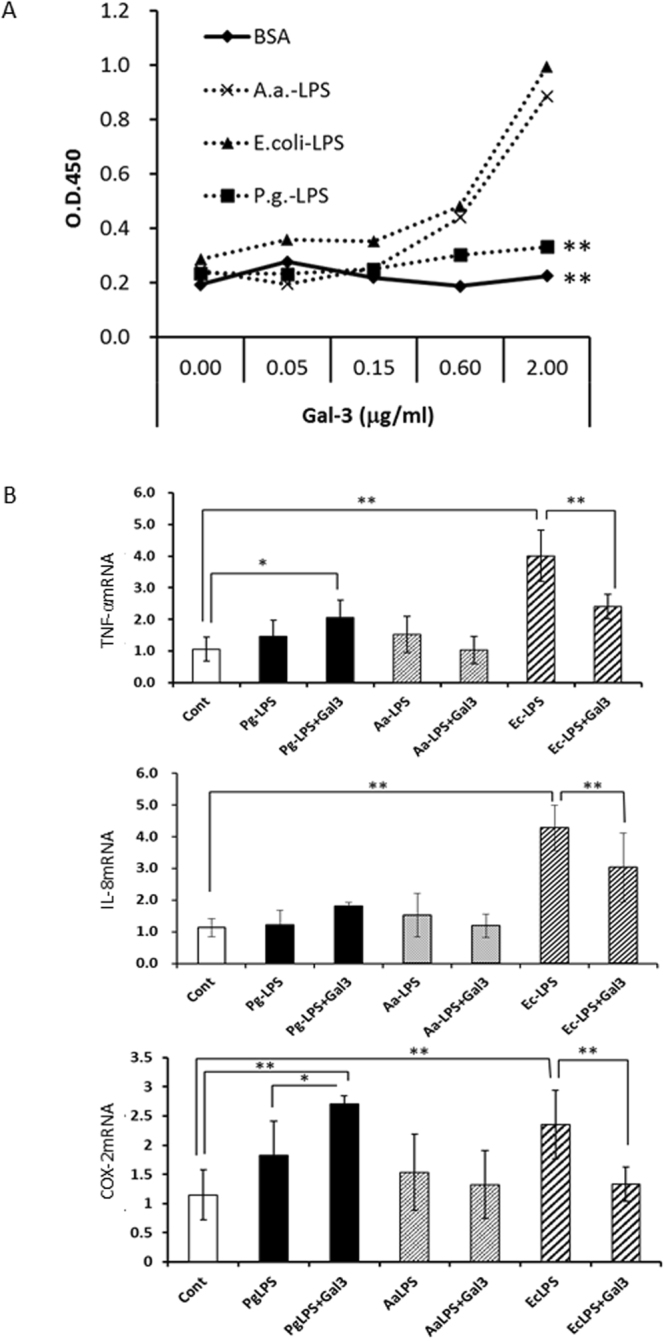
Gal-3 did not bind to *P.g.-*LPS, and both molecules synergistically upregulated the expression of inflammatory mediators. (**A**) Studying the binding affinity of Gal-3 for LPS from *A.a., E. coli*, or *P.g*., as described in the Material and Methods section. *P.g*.-LPS did not bind Gal-3. (**B**) Trophoblasts (HTR-8 cells) were stimulated for 48 h with LPS (100 ng/ml) from *P.g*., *A.a*., or *E. coli* with or without rhGal-3 (0.5 µg/ml). Synergistical upregulation of cytokine mRNA expression was evident by *P.g-* LPS. but not by *A.a*. or *E. coli-*LPS using the real time PCR analysis. GAPDH was used as an internal control. Data are presented as the mean ± SD. *P < 0.05; **P < 0.01; 1-way ANOVA. Experiments were performed at least three with similar results.

Gal-3 can bind LPS and might inhibit LPS-induced proinflammatory responses^20^. Here, a binding assay was performed to examine the relationship between LPS and Gal-3. Gal-3 could bind to LPS from *A.a*. and *E. coli*; however, *P.g*.-LPS and Gal-3 did not bind each other (Fig. 6A). Co-stimulation of rhGal-3 and *P.g*.-LPS upregulated COX-2, and TNF-α mRNA expression, compared with their levels of *P.g*.-LPS treatment alone (Fig. 6B, supplement Fig. 2A). In contrast, co-stimulation of rhGal-3 and *E. coli-*LPS significantly downregulated TNF-α, IL-8 and COX-2 versus LPS treatment alone. Co-stimulation of rhGal-3 and *A.a.-*LPS also showed a tendency to downregulate these molecules (Fig. 6B, Supplement Fig. 2A).

## Discussion

In this study, we used a mouse model of *P.g*.-dental infection. *P.g*., which is a main causative pathogen of chronic and periapical periodontitis, is infected from the pulp chamber because it requires anaerobic growth conditions^21^. The pulp chamber provides ideal anaerobic conditions and is the natural infecting way of periapical periodontitis. Previously, we demonstrated that *P.g*. remained alive inside the pulp chamber, reproduced long enough to induce periapical periodontitis, and maintained upregulated LPS levels in the serum^22^. Moreover, we confirmed the establishment of chronic, low-grade systemic inflammation, including upregulated TNF-α, IL-17, IL-6, and IL-1β serum levels at 6-weeks post-*P.g*. infection, the time of mating^16^. These data indicated that the mouse model successfully mimicked the clinical conditions of pregnant women with chronic dental infection. We also reported previously that *P.g*.-infected mice showed PTB by 2 days, on average^16^, which was comparable with that observed by direct intrauterine injection of *E. coli* (10^4^ CFU)^23^.

Interestingly, Gal-3, TNF-α and COX-2 immunoexpression dramatically increased in *P.g*.-infected placenta in this study. The Gal-3 level in the amniotic fluid and serum was also significantly upregulated in *P.g.-*infected mice (Fig. 2). Gal-3, a beta-galactoside-binding lectin, plays both pro-inflammatory and anti-inflammatory roles in the inflammation process^24^. Extracellular Gal-3 contributes to neutrophil adhesion, macrophage migration, and endocytosis, indicating the pro-inflammatory roles of Gal-3. Therefore, it is possible that extracellular Gal-3 may play an important role in the onset of PTB. However, to our knowledge, the relationship between Gal-3 and PTB has not yet been elucidated.

It is well accepted that bacterial LPS contributes to pathogenesis of PTB through TLR4^25^. LPS is a main virulence factor of *P.g*., and elevated LPS levels in the sera of *P.g*.-infected mice were observed in a previous study^22^. We reported that TNF-α, IL-8 and COX-2 expression, which relate to onset of PTB, were secreted from HTR-8 cells with *P.g*.-LPS stimulation^16^. Ren *et al*. also demonstrated that *P.g*.-LPS promoted IL-8 secretion from HTR-8 cells^26^.

*P.g*.-LPS stimulation increased Gal-3 expression in placental cells (trophoblasts) by activating the NF-κB and MAPK signaling pathways (Fig. 3A,C). Moreover, exogenously applied rhGal-3 or rhTNF-α, which were produced by LPS-stimulated trophoblasts, also increased Gal-3 production. It was previously reported that extracellular Gal-3 modulated the production of cytokines such as IL-1β^27^, IL-5^28^, and IL-8^29^ not only in immune cells, but also in another cell types^30^. In the present study, Gal-3 was also responsible for upregulating proinflammatory mediators, such as COX-2, IL-8, and TNF-α. Gal-3 and TNF-α, which were produced by *P.g*.-LPS stimulation in *P.g*.-infected placenta, induced additional Gal-3-production, finally leading to excessive cytokine expression. It was suggested that in placenta of *P.g*.-odontogenic infected animals, harmful cycle in cytokine through Gal-3 may be established. Moreover, inhibition of Gal-3 by Gal-3 neutralizing antibody significantly suppressed *P.g*.-LPS induced TNF-α production, resulting in reduction of IL-8 and COX-2. We consider that the regulation of Gal-3 in placenta may be a possible preventive strategy of PTB.

Our previous report showed that immunohistochemically *P.g*. detected in the placental cells (trophoblasts) of *P.g*.- dental infection mouse. *In vitro* study showed that HTR-8 cells with *P.g*.-infection increased secretion of COX-2, TNF-α, and IL-8^16^. Several reports indicated that infection of trophoblasts by *P.g*. induced cytokine expression^26,31^ and apoptosis^32,33^ through MAP kinase signalling^34^. It is indicating that *P.g*.-infection to trophoblasts also has impacts on pathogenesis of PTB caused by periodontitis.

In contrast, it was also reported that Gal-3 shows an anti-inflammatory effect by binding to LPS. Mey *et al*.^35^ reported that Gal-3 binds to LPS from gram-negative bacteria, and Li *et al*. showed the inhibitory effect of Gal-3 in *E. coli-*LPS-induced inflammation^20^. We examined the binding affinity between Gal-3 and various types of LPS. Here, we found that Gal-3 bound to LPS from *E. coli* and *A.a*., but not *P.g*.-LPS, in agreement with the report by Kato *et al*.^36^. LPS consists of a hydrophobic lipid embedded in the outer leaflet of the outer membrane, a core oligosaccharide (OS), and the O-polysaccharide side chain composed of several repeating units^37^. It was reported that heptose residues in core OS of LPS provided the attachment point with various types of LPS. Using heptose mutation, Quattroni *et al*. found that heptose in the core OS in *Neisseria meningitidis* was necessary for the Gal-3 binding to LPS^38^. The LPS core OS molecules of *E. coli* and *A.a*. contain heptose residues^39^, whereas *P.g*.-LPS lacks heptose residues^37,40^. Co-stimulation of rhGal-3 and *E. coli-*LPS or *A.a.-*LPS significantly downregulated cytokine levels versus LPS treatment alone. However, since the anti-inflammatory effect of Gal-3 is limited in early stage of LPS stimulation, the prolonged and excessive production of Gal-3 finally may induce proinflammatory effect. On the other hand, co-stimulation with rhGal-3 and a low dose of *P.g.-*LPS revealed a synergistic effect on the upregulation of inflammatory mediators. Since *P.g*.-LPS can synergistically induce intense cytokine production in early stage of placental inflammation, in which abundant Gal-3 present, even *P.g* with low virulence LPS than *E. coli*, can be a risk for PTB.

Our investigation showed that Gal-3 inhibited CD66a expression. CD66a is an adhesion molecule of the carcinoembryonic antigen family. Bamberger *et al*.^18^ reported that CD66a was strongly expressed by intermediate trophoblasts, endometrial epithelial cells, and endothelial cells at the maternal-fetal interface throughout pregnancy. Thus, CD66a may play an important role in adhesive trophoblast-endometrial and trophoblast-endothelial cell interactions. CD66a downregulation by Gal-3 may induce cell adhesion breakdown at the maternal-fetal interface and contribute to the initiation of placental abruption from the uterus.

Moreover, we clarified that Gal-3 levels in the amniotic fluid and maternal serum of *P.g.-*infected mice were significantly higher than those of NC mice, indicating that the Gal-3 level may be potent predictive biomarker for PTB. Predictive biomarkers for PTB are urgently needed to reduce PTB caused by maternal infection/inflammation during the gestation period. Several inflammatory biomarkers present in amniotic fluid have been proposed as predictors of intra-amniotic inflammation and preterm labour. IL-6 has shown the best sensitivity for predicting infection in patients with preterm labour^7,41^. Recently, the combined use of IL-6 and proteomic biomarkers was recommended^42^. However, their clinical use is still limited. As mentioned above, we demonstrated significant Gal-3 elevation in the amniotic fluid of *P.g*. dentally infected mice. Gal-3 in the amniotic fluid may be an important predictive marker for PTB. However, examination of the amniotic fluid is invasive testing that causes an increased risk of abortion. Non-invasive diagnostic testing is key for screening pregnant individuals. Maternal serum is easily collected without threatening the pregnancy. In this study, we also detected Gal-3 upregulation in the maternal serum. This is the first investigation of the association of serum Gal-3 upregulation with PTB. In human serum, Gal-3 is constitutively detected with mean serum levels of approximately 5 ng/ml in healthy adults^43^. Increase serum Gal-3 is associated with various pathological conditions, such as immune responses^44^ and malignancy^24^. Demmert *et al*.^45^ demonstrated that the Gal-3 level was significantly higher in cord blood of small-for-gestational-age infants. However, there is still no report showing a relationship between maternal serum Gal-3 levels and PTB. In the present study, we detected significant upregulation of the serum Gal-3 level at 15gd in *P.g*. dentally infected mice with PTB. Serum Gal-3 may serve as a novel predictive biomarker for PTB, although further clinical studies are needed to clarify the usefulness of Gal-3 upregulation in the amniotic fluid and/or serum as a predictive marker of PTB.

**Figure 7:**
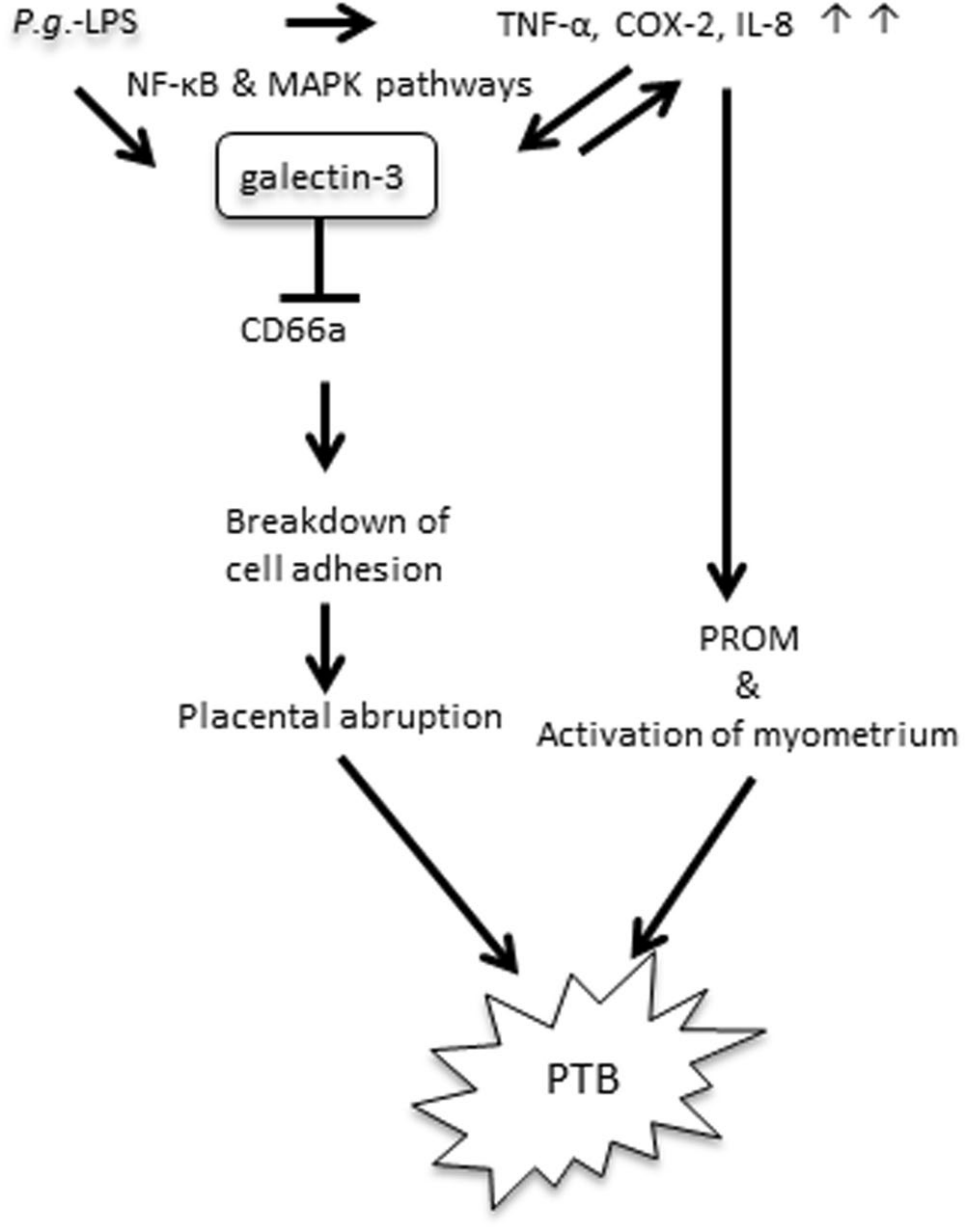
The possible mechanisms responsible for *P.g*. dental infection induced PTB through Gal-3. Dental infection of *P.g*. increases *P.g*.-LPS and inflammatory cytokines in serum. In placenta, *P.g*.-LPS activates NF-κB and MAPK pathways and induces production of TNF-α, IL-8 and COX-2 that lead to premature rupture of membrane (PROM) and activation of myometrium. Moreover, *P.g*.-LPS and cytokines produced by *P.g.-*LPS stimulation upregulate Gal-3. The produced Gal-3 may also contribute to excessive production of cytokines. Eventually, excessively produced cytokines may result in PTB. Alternatively, *P.g*.-LPS induced Gal-3 may inhibit CD66a. Breakdown of cell adhesion at maternal-infant interface leads to placental abruption and PTB.

Based on our results, we considered possible mechanisms responsible for *P.g*. dental infection-induced PTB through Gal-3 (Fig. 7). Dental *P.g*. infection increases inflammatory cytokines in the serum and induces *P.g.-*LPS translocation in the placenta through the blood circulation. In the placenta, *P.g*.-LPS upregulated inflammatory mediators such as TNF-α, IL-8, and COX-2 directly or indirectly through Gal-3 production. Upregulated TNF-α can also induce Gal-3 production, which may help establish the excessive production of pro-inflammatory mediators through Gal-3. Gal-3 inhibits CD66a expression, which results in the breakdown of cell adhesion and induces placental abruption. In addition, the regulation of inflammatory mediators induces cervical ripening and activation of the myometrium. Taken together, the findings indicate that Gal-3 plays an important role in PTB induced by dental infection with *P.g*. Thus, dental treatments to prevent and/or eliminate *P.g*. infection should be meticulously performed throughout the world to reduce the PTB rate. Gal-3 should be considered as one of the PTB biomarkers in the amniotic fluid and/or serum, and should be considered as a possible new target for preventing PTB.

## Materials and Methods

### Animal Model

This study was performed in strict accordance with the recommendations in the Guide for the Care and Use of Laboratory Animals of the Hiroshima University Animal Research Committee and AVMA Guidelines on Euthanasia. The protocol (Supplementary Fig. 3) described below was approved by the Committee on the Ethics of Animal Experiments of the Hiroshima University (Permit Number: A09-89). Eight-week-old female C57Bl/6J mice (Charles River Japan, Inc., Yokohama, Japan) were housed in a specific-pathogen free facility in 12-h light-dark cycles with access to water and food *ad libitum*, and health monitoring was conducted every day. Mice were randomly divided into 2 groups with or without *P.g*. infection and designated as the *P.g*.-infected group or the non-infected control (NC) group, respectively. *P.g*. dental infection was performed as described previously^16,21^. Briefly, the pulp chambers of the upper first molars on both sides were opened with a #1/2 round burr. After removing the coronal pulp, a small cotton swab containing 10^8^ colony-forming units (CFU) of *P.g*. (W83 strain) was inserted into the pulp chamber and sealed with Caviton (GC Co., Tokyo, Japan). At 6 weeks post-*P.g*. infection, mating was started. Placental tissues, amniotic fluid, and serum were harvested at 15 gestational day (gd).

### Histology

Placental tissues were fixed in 10% neutral buffered formalin for 24 h. Samples were processed, embedded in paraffin, and serial sections of 4.5 μm in thickness were obtained for hematoxylin and eosin (HE) staining and immunohistochemistry, which was performed as described previously^16^. Staining was visualized using the DAB Peroxidase (HRP) Substrate Kit (Dako Japan, Tokyo, Japan) to produce a brown reaction product. Immunohistochemistry were performed using high polymer method (Histofine Max-PO; Nichirei Biosciences). A primary Gal-3 antibody (BioLegend, Inc., San Diego, CA, USA; 1: 500) was used. Specificity was ascertained by substituting PBS/serum for the antibody.

### Cell Lines and Cell Culture

HTR-8/Svneo cells (human trophoblast cell line) were kindly provided by Professor Charles H. Graham (Queen’s University, Canada). The cells were grown in RPMI 1640 media (Nissui Pharmaceutical Co., Tokyo, Japan) supplemented with 10% heat-inactivated foetal bovine serum (Invitrogen) and 100 U/ml penicillin–streptomycin (Gibco, Tokyo, Japan). For the experiments, cells were seeded at a density of 5 × 10^5^ cells in 35-mm culture dishes. The cells were maintained at 37 °C in a normal atmosphere containing 5% CO_2_.

### Enzyme-Linked Immunosorbent Assay (ELISA)

Mouse Gal-3 concentrations in amniotic fluids and sera were measured using the Galectin-3 DuoSet ELISA Development Kit (R&D Systems, Minneapolis, MN, USA) according to the manufacturer’s instructions. *P.g*.-LPS, which is a TLR2 and TLR4 ligand, was purchased from InvivoGen (San Diego, CA, USA). HTR-8 cells were stimulated with *P.g.-*LPS, recombinant human Gal-3 (rhGal-3; PeproTech, Rocky Hill, NJ, USA), or recombinant human TNF-α (rhTNF-α; R & D Systems), as indicated below, and the protein levels of Gal-3 or TNF-α in cell culture supernatants were analysed using the Human Galectin-3 Immunoassay (R & D Systems) or the Human TNF-α Immunoassay (R & D Systems). Culture supernatants from *P.g*.-LPS-stimulated HRT-8 cells, grown in the presence or absence of dicumarol (a JNK MAPK inhibitor; 10 μM), SB203580 (a p38 MAPK inhibitor; 10 μM), U0126 (an ERK1/2 MAPK inhibitor; 10 μM) or caffeic acid phenethyl ester (CAPE; an NF-kB inhibitor; 10 μg) (Sigma-Aldrich) were also analysed.

### RNA Isolation from Placental Tissues/Cells and RT-PCR Analysis

Total RNA was extracted using the RNeasy Mini Kit (Qiagen, K.K., Tokyo, Japan) following the manufacturer’s instructions. The RNA concentration and purity were determined using standard spectrophotometric methods. One microgram of total RNA was used for cDNA synthesis using the ReverTra Dash Kit (TOYOBO, Osaka, Japan). Total cDNA was amplified using the Go Taq Green Master Mix (Promega, Madison, WI, USA). Amplification of human or mouse COX-2, Gal-3, IL-8/mKC, and TNF-α was performed in a MyCycler^TM^ thermal cycler (Bio-Rad, Tokyo, Japan) for 30 cycles of denaturation for 30 s at 94 °C, annealing for 30 s at 58 °C, and extension for 1 min at 72 °C, using primers for all each target mentioned above. GAPDH expression was detected as an internal control. PCR primer sequences are listed in Supplemental Table 1. The amplification products were resolved on 1.5% agarose/TAE gels (Nacalai Tesque, Inc., Kyoto, Japan), electrophoresed at 100 mV, and visualized by ethidium-bromide staining.

Quantitative real time PT-PCR was performed in the Applied Biosystem StepOnePlusTM (Applied Biosystems, http://www.appliedbiosystems.jp) using TaqMan® Fast Advanced Master Mix (Applied Biosystems) and specific primers and probe. Specific primers and probe for mTNF-α (Forward: CCAAATGGCCTCCCTCTCAT, Reverse; GCTACAGGCTTGTCACTCGAATT, Probe; CCCAGACCCTCACACTCAGATCATCT) and hTNF-α (Forward: CCTGCCCCAATCCCTTTATT, Reverse; CCAATTCTCTTTTTGAGCCAGAA, Probe; CCCCCTCCTTCAGACACCCTCAACC) were used. Other sets of specific primers and probe for mGal-3 (Mm00478303), mCOX-2 (Mm03294838), mIL-8(Cxcl15; Mm00441263), mGAPDH (Mm03302249), hGal-3 (Hs00174774), hCOX2 (PTGS2; Hs00153133), hIL-8 (CXCL8; Hs00174103) and hGAPDH (Hs02786624) were purchased from Applied Biosystems. The reaction product was quantified with each GAPDH as the reference gene.

### ELISA Analysis for Binding of Gal-3 to LPS

Experiments was performed as described previously, with slight modifications^36^. The 96-well ELISA plates (Costar High binding 3590, Fisher Scientific, Japan) were coated with *Aggregatibacter actinomycetemcomitans-*LPS (*A.a.-*LPS) kindly provided by Dr. Tatsuji Nishihara of Kyushu Dental College, Japan), *P.g.-*LPS, *Escherichia coli*-LPS (*E. coli-*LPS; Wako Pure Chemical Industries, Ltd., Ohsaka, Japan) (50 μg/ml each), or 3% bovine serum albumin (BSA; as a control) and incubated overnight at 4 °C. Plates were washed twice with ELISA buffer (Tris-buffered saline with 0.05% Tween 20, 1 mM CaCl_2_, and 0.1% BSA, pH 7.4), and 3% Gelatin was used for blocking at 37 °C for 1 h. rhGal-3 was added to each well at different final concentrations (0, 0.05, 0.15, 0.6, or 2 μg/ml) and incubated overnight at 4 °C. An anti-Gal-3 antibody (BioLegend, Inc.) was also added to each well, and the plates were incubated overnight at 4 °C. For detection, a horseradish peroxidase-conjugated anti-rat IgG (GE Healthcare Life Sciences) was added, and tetramethyl benzidine substrate (colour reagent A & B, 1: 1, R&D Systems) was used for visualization. After 20 min, stop solution (R&D Systems) was added. O.D.s were measured at 450 nm.

### Small interfering RNA (siRNA)-mediated Gal-3 Gene Knockdown

siRNAs against human Gal-3 were purchased from Bonac Corporation (Fukuoka, Japan). The siRNA sequences are shown in Table 1. Transfection was performed using Lipofectamine RNAi Max (Invitrogen), according to the manufacturer’s recommended protocol.

**Table 1:** Sequences of primer pairs.

Gene	Forward	Reverse	Size	Accession number
**Mouse**				
COX-2 (Ptgs2)	TTCAAAAGAAGTGCTGGAAAAGGT	GATCATCTCTACCTGAGTGTCTTT	304	NM_011198.3
mKC (IL-8 homologue)	CGCTGCTGCTGCTGGCCACCA	GGCTATGACTTCGGTTTGGGTGCAG	164	NM_008176.3
TNF-α	ATGAGCACAGAAAGCATGATC	TACAGGCTTGTCACTCGAATT	276	NM_013693.2
GAPDH	GCATCCTGGGCTACACTGAG	TCCACCACCCTGTTGCTGTA	163	NM_008084.2
**Human**				
COX-2 (Ptgs2)	TGAGCATCTACGGTTTGCTG	TGCTTGTCTGGAACAACTGC	158	NM_000963.2
IL-8	TAGCAAAATTGAGGCCAAGG	GGACTTGTGGATCCTGGCTA	204	NM_000584.3
TNF-α	AAGAATTCAAACTGGGGCCT	GGCTACATGGGAACAGCCTA	402	NM_000594.2
GAPDH	TGAACGGGAAGCTCACTGG	TCCACCACCCTGTTGCTGTA	307	NM_002046.4
**siRNA**				
siGal-3	CACGCUUCAAUGAGAACAACA	UGUUGUUCUCAUUGAAGCGUG		

### Western Blotting

Western blotting was performed as previously described^46^. Briefly, HTR-8 cell pellets (5 × 10^5^ cells) were resuspended in ice-cold lysis buffer. Proteins were separated by sodium dodecyl sulphate-polyacrylamide gel electrophoresis and electro-blotted onto a nitrocellulose membrane. After blocking the membrane in 3% milk for 30 min, immunoblotting was performed using primary antibodies (anti-phospho-JNK, anti-phospho-c-Jun, anti-phospho-ERK1/2, anti-ERK1/2, anti-phospho-NF-κB p65, anti-RelB [Cell Signaling Technology], or anti-CD66a [Epitomics, Burlingame, CA, USA]) and secondary antibodies (GE Healthcare Life Science, Tokyo, Japan), as indicated. β-actin (Sigma-Aldrich, St. Louis, MO, USA) was detected as an internal control. The results were visualized using the ECL Western Blotting Detection System (GE Healthcare, UK).

### Statistical Analysis

Data are presented as the mean ± standard deviation. Statistical differences among experimental groups were evaluated by 1-way analysis of variance (ANOVA), followed by Tukey’s post-hoc test or Student’s t-test using GraphPad Prism (version 6.0c, CA, USA) with the levels of significance set at ^*^P < 0.05 and ^**^P < 0.01.

### Data availability

All data generated or analysed during this study are included in this published article and its Supplementary Information files.

## Electronic supplementary material

Supplementary Information
