# Supplementary material for: Galectin-3 Plays an Important Role in Preterm Birth Caused by Dental Infection of Porphyromonas gingivalis
Source: Sci Rep. 2018 Feb 12;8:2867. doi: 10.1038/s41598-018-21072-y (PMC5809409; doi:10.1038/s41598-018-21072-y)
Supplement: Supplementary file 1 — Supplementary Information [file 41598_2018_21072_MOESM1_ESM.pdf]

Galectin-3 Plays an Important Role in Preterm Birth Caused by Dental Infection of *Porphyromonas gingivalis*

Mutsumi Miyauchi[1*], Min Ao[1,2*†], Hisako Furusho[1*], Chanbora Chea[1], Atsuhiro Nagasaki[1], Shinnichi Sakamoto[1], Toshinori Ando[1], Toshihiro Inubushi[1††], Katsuyuki Kozai[2] and Takashi Takata[1]

Supplemental Figure 1

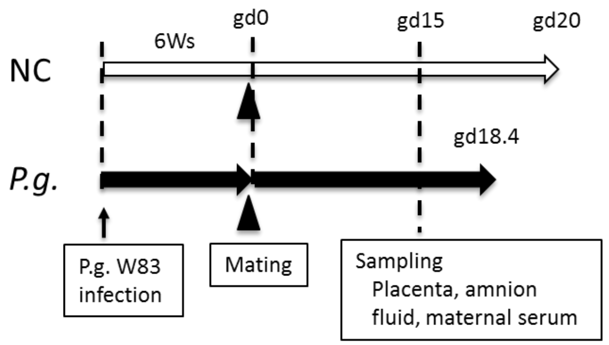

Supplementary Figure 1
Gal-3mRNA expression in macrophages and endothelial cells.
Gal-3 mRNA expression and production from macrophage (PMA stimulated THP-1) (A) and endothelial cell (HuhT1) (B) with *P.g.*-LPS (1μg/ml) stimulation. Data are presented as the mean ± SD. **P < 0.01; Student's t-test.

Supplemental Figure 2

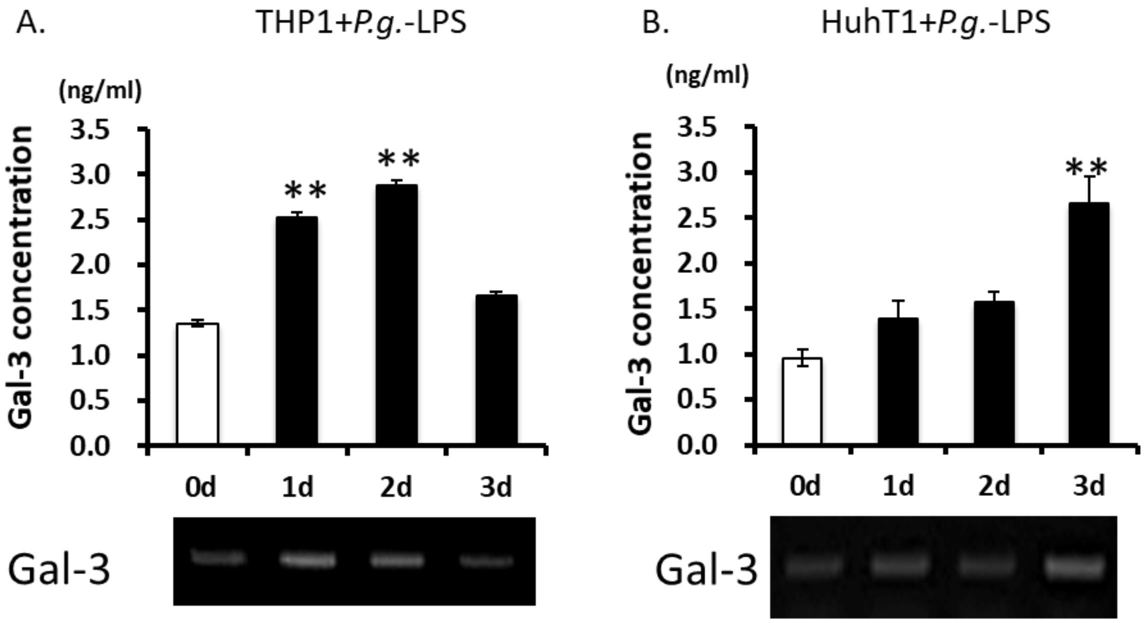

Supplementary Figure 2

Effects of costimulation of Gal-3 and various types of LPS.

Trophoblasts (HTR-8 cells) were stimulated for 48 hours with LPS (100 ng/ml) from P.g. (A), A.a., or E. coli (B) with or without rhGal-3 (0.5 µg/ml). COX-2, IL-8 and TNF-α mRNA expression was analysed by the PCR. Synergistically upregulated cytokine mRNA expression was seen by combination with *P.g*- LPS and Gal-3. The combination with *A.a.* or *E. coli*-LPS showed reduced expression of them. GAPDH was used as an internal control.

Supplemental Figure 3

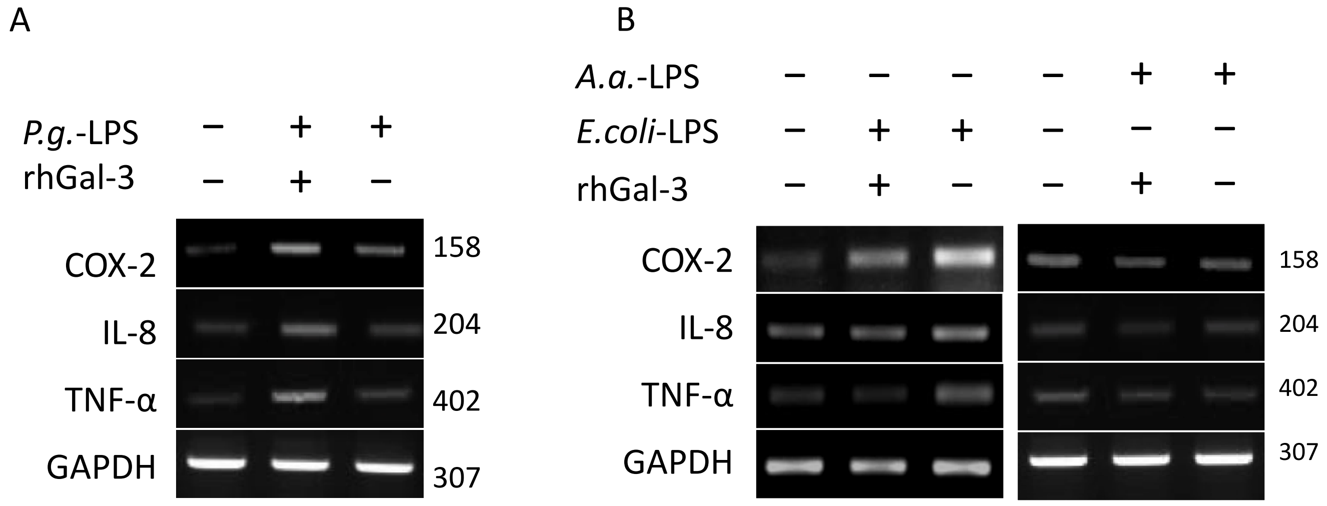

Supplementary Figure 3.
Experimental schedule to assess the effects of dental *P.g.* infection on placental tissues.
